# Selective retina therapy monitoring by speckle variance optical coherence tomography for dosimetry control

**DOI:** 10.1117/1.JBO.25.2.026001

**Published:** 2020-02-14

**Authors:** Soohyun Lee, Shuwen Wei, Shoujing Guo, Jongmin Kim, Bongkyun Kim, Gihoon Kim, Jin U. Kang

**Affiliations:** a Johns Hopkins University, Department of Electrical and Computer Engineering, Baltimore, Maryland, United States; b Lutronic Center, Goyang, Republic of Korea

**Keywords:** biomedical optics, speckle variance optical coherence tomography, selective retina therapy, ophthalmology

## Abstract

**Significance:** Selective retina therapy (SRT) selectively targets the retinal pigment epithelium (RPE) and reduces negative side effects by avoiding thermal damages of the adjacent photoreceptors, the neural retina, and the choroid. However, the selection of proper laser energy for the SRT is challenging because of ophthalmoscopically invisible lesions in the RPE and different melanin concentrations among patients or even regions within an eye.

**Aim:** We propose and demonstrate SRT monitoring based on speckle variance optical coherence tomography (svOCT) for dosimetry control.

**Approach:** M-scans, time-resolved sequence of A-scans, of *ex vivo* bovine retina irradiated by 1.7-μs duration laser pulses were obtained by a swept-source OCT. SvOCT images were calculated as interframe intensity variance of the sequence. Spatial and temporal temperature distributions in the retina were numerically calculated in a 2-D retinal model using COMSOL Multiphysics. Microscopic images of treated spots were obtained before and after removing the upper neural retinal layer to assess the damage in both RPE and neural layers.

**Results:** SvOCT images show abrupt speckle variance changes when the retina is irradiated by laser pulses. The svOCT intensities averaged in RPE and photoreceptor layers along the axial direction show sharp peaks corresponding to each laser pulse, and the peak values were proportional to the laser pulse energy. The calculated temperatures in the neural retina layer and RPE were linearly fitted to the svOCT peak values, and the temperature of each lesion was estimated based on the fitting. The estimated temperatures matched well with previously reported results.

**Conclusion:** We found a reliable correlation between the svOCT peak values and the degree of retinal lesion formation, which can be used for selecting proper laser energy during SRT.

## 1 Introduction

Selective retina therapy (SRT) is an effective laser treatment method for various retinal diseases associated with degradation of the retinal pigment epithelium (RPE), such as diabetic macular edema, central serous chorioretinopathy, and age-related macular degeneration.^1^^–^^6^ The RPE, which contains a high concentration of melanosomes, absorbs 50% to 60% of incident green light. However, in order to selectively target the RPE layer, the laser pulse duration needs to be shorter than a thermal relaxation time of the RPE (∼10  μs).^7^ The SRT reduces negative side effects and facilitates healing of the induced retinal lesions by avoiding thermal damages of the adjacent photoreceptors, the neural retina, and the choroid. However, the selection of proper laser energy—which is crucial for successful SRT without excessive burning and collateral damage—is challenging because lesions in the RPE are ophthalmoscopically invisible. In addition, different melanin concentrations among patients, or regions even within an eye,^8^ make it impossible to set a static threshold value of pulse energy of a therapeutic irradiation window.

Fundus fluorescence angiography (FFA) is an accurate method to detect the lesions, but it requires the use of fluorescent dye injection^9^ and a long delay between treatment and detection. For real-time noninvasive SRT monitoring, several approaches have been proposed. These include the detection of microbubble formation and collapse, which induce mechanical disruption and damage to RPE cells.^10^ This approach measures the acoustic transient^11^ or light reflection changes.^12^ Although these methods have already been used in several clinical studies,^11^^,^^13^^,^^14^ they do not provide visual feedback during the treatment. Optical coherence tomography (OCT), which can provide depth-resolved imaging, was also applied for the SRT monitoring.^15^^–^^19^ The treatments are considered successful when OCT signal variations, i.e., intensity decrease, are detected, and the results show good agreement with the evaluation of lesions by FFA.

Speckle variance OCT (svOCT) quantifies the speckle pattern variation caused by moving particles or structural changes in biological tissues. It calculates the interframe intensity variance of a sequence of structural OCT images. The svOCT has been extensively developed in recent years for OCT angiography, which is used to visualize retinal microvasculatures;^20^ it has also been shown to be able to monitor protein denaturation and coagulation.^21^ Thus, it is expected that svOCT could be an effective way to detect speckle variation changes induced by morphological and structural changes of retinal tissue during the thermal-induced microbubble formation and collapse by laser irradiation. In this paper, we studied and demonstrated SRT monitoring based on the svOCT. A swept-source OCT imaging system integrated with a microsecond pulsed laser system was used for *ex vivo* bovine retina study. SvOCT images corresponding to various laser pulse energies and the various number of frames were obtained. The svOCT values of RPE and photoreceptor layers were averaged along the axial direction, and peak values of the svOCT at each pulse laser irradiation were analyzed. The microscopic images of the treated spots were taken before and after removing the upper neural retinal layers to assess the degree of retina and RPE damage. Spatial and temporal thermal effects in retina induced by pulse laser irradiation were simulated and correlated to the peak values of svOCT.

## 2 Experimental Method

**Fig. 1:**
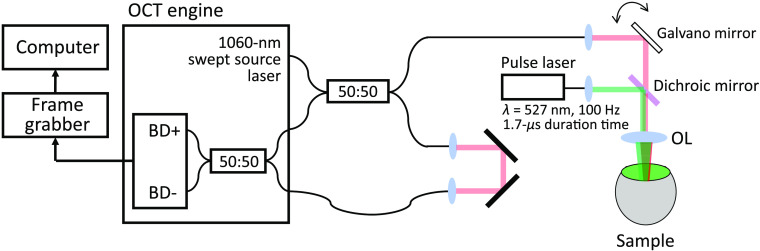
Schematic of a swept-source OCT system integrated into a pulse laser system. BD, balanced detector; OL, objective lens.

An in-house built swept-source OCT imaging system was integrated with a frequency-doubled Nd:YLF laser-based SRT system (Lutronic, Goyang, Korea). The schematic of the system is shown in Fig. 1. The OCT system used a commercial swept-source engine (Axsun Technologies Inc., Billerica, Massachusetts) operating at 100-kHz sweep rate. The center wavelength and sweeping bandwidth of the system were 1060 and 100 nm, respectively. The OCT laser was combined with the pulse laser using a dichroic mirror; galvano mirrors were used to direct the OCT laser to a treated spot on the retina. The wavelength of the pulse laser was 527 nm, and the pulse laser operated at 100-Hz repetition rate and 1.7-μs duration. The pulse laser energy was adjusted from 22 to 190  μJ using neutral-density filters. In this study, fresh *ex vivo* bovine eyes were acquired from a local butcher and immersed in a cooled saline solution. Bovine eyes have tapetum fibrosum, which has the retinal epithelial layer completely unpigmented, over the central and mid-region of the retina.^22^ Because the RPE layer exists in the periphery of the retina, it was difficult to focus the beam on the RPE layer using the crystalline lens of an eye itself. Therefore, the bovine eye’s cornea and lens were removed, and the beam was focused on a tilted eye using an objective lens. A total of 39 treated spots were tested on two eyes. M-mode OCT images of the bovine retina were acquired during the laser pulse irradiation.

SvOCT images were calculated by

$$SV_{ij}=\frac{1}{N}\overset{i*N}{\underset{k=(i-1)*N+1}{\sum }}{\left[I_{k}(j)-\frac{1}{N}\overset{i*N}{\underset{l=(i-1)*N+1}{\sum }}I_{l}(j)\right]}^{2},$$

where i, j, and k are the indices of the frame of the svOCT images, axial position, and the frame of M-scans, respectively, and N is the number of frames used for variance calculation. The photoreceptor and RPE layers, which are highly scattering and absorptive, were set as a region of interest (ROI), and the svOCT values in the ROI were averaged along the axial direction. Microscopic images of the treated spots were obtained using a CCD camera (DCC1645C, Thorlabs, Newton, New Jersey) and a 10× magnification zoom lens.

**Fig. 2:**
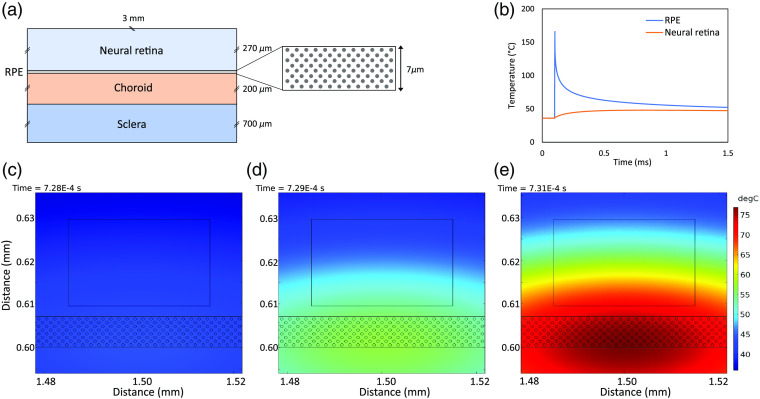
(a) The geometry of the bovine retina model. The retina was assumed to consist of two layers, neural retina and RPE, and has immediate contact with a choroid. The RPE was modeled as a 7-μm layer containing melanosomes that were assumed as diagonally distributed spheres of radius 0.3  μm. (b) Temperature–time dependence in the neural retina and at the melanosome surface in RPE when laser pulse of energy 50  μJ irradiated. Spatial distribution of temperature around RPE, when the temperature of the neural retina reached a maximum after (c) 20-μJ, (d) 50-μJ and (e) 100-μJ pulse irradiations.

Spatial and temporal temperature distributions in the retina were numerically calculated by COMSOL Multiphysics software. Spatial distribution was calculated in 2-D, and the geometry of the bovine retina model used for the simulation is shown in Fig. 2(a). The RPE was modeled as a 7-μm layer containing melanosomes that were assumed as spheres of radius 0.3  μm by a discrete absorber model.^23^ Melanosomes were diagonally distributed, and the distance between adjacent melanosomes was set to 1.2  μm. The model applied a heat equation as shown below:

$$\rho C_{p}\frac{\partial T}{\partial t}=\nabla \cdot (k\nabla T)+Q,$$

where T is the temperature as a function of time (t) and spatial coordinate x and y, ρ is the density, k is the thermal conductivity, and $C_{p}$ is the heat capacity of the material. The Q refers to the heat source from laser irradiation. The coefficient values used are shown in Table 1, which include the thickness, absorption coefficient, and thermal physical constant values^25^ of each retinal layer. The absorption coefficient of retinal melanosomes at 532-nm wavelength was estimated from 2370 to $13000\;{\mathrm{cm}}^{-1}$,^10^^,^^26^ and $6500\;{\mathrm{cm}}^{-1}$, which makes the absorption in RPE around 50% of the total, was used for our simulation. The duration and frequency of the pulse laser were set to 2  μs, 5  μs or 10  μs and 100 Hz, respectively; the peak temperature was calculated over 1.5 ms. Gaussian laser-beam profile with a diameter of 150  μm was used for the simulation.

**Table 1:** Thickness, absorption coefficient, and thermal properties of each retinal layer.

	Neural retina	RPE layer outside of melanosome	Melanosome	Choroid	Sclera
Thickness (μm)	270	7	0.3 (radius)	200	700
Absorption coefficient (${\mathrm{cm}}^{-1}$)	10.4^24^	0	6500	245^24^	4.9
Heat capacity (J/kg·K)	3680	3680	3680	3680	4178
Density ($\mathrm{kg}/m^{3}$)	1000	1000	1000	1000	1000
Thermal conductivity (W/m·K)	0.565	0.565	0.565	0.530	0.58

The temperature of the neural retina was calculated by averaging the temperature in a rectangular region (30  μm×20  μm) 2.5  μm away from RPE, and the temperature of the melanosome surface in RPE was calculated by averaging the temperature on seven melanosome surfaces at the first two melanosome layers located in the center of the Gaussian beam. Figure 2(b) shows the time-dependent temperature variation in the neural retina (in orange) and at the melanosome surface in RPE (in blue). Spatial distributions of temperature around RPE, when the temperature of neural retina reached a maximum after a 20-, 50-, and 100-μJ pulse irradiation, are shown in Figs. 2(c)–2(e). The calculated peak temperatures in each region were correlated to the peak values of svOCT and tissue damage.

## 3 Results

**Fig. 3:**
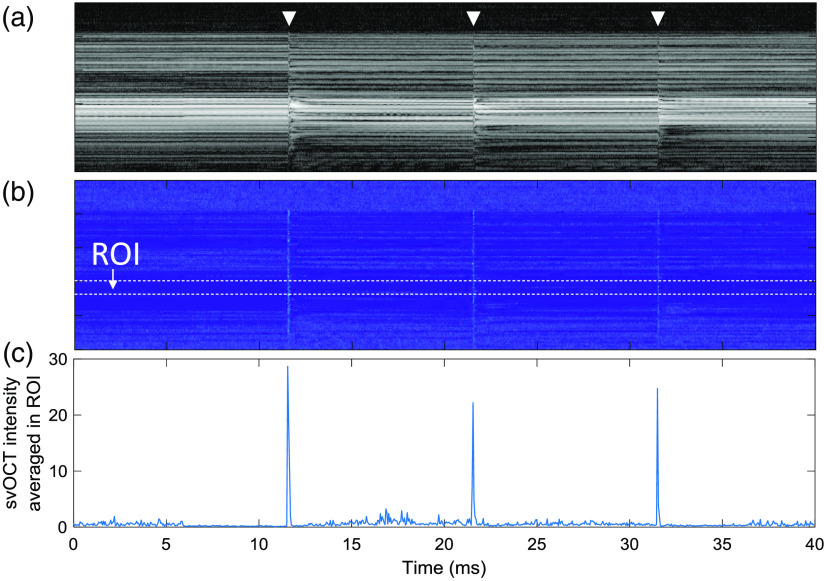
(a) M-mode OCT image of the bovine retina and corresponding (b) svOCT image. Photoreceptor and RPE layers, which are highly scattering and absorptive, were set as an ROI. (c) Axially averaged svOCT values in the ROI during pulse laser irradiation. White triangles mark the moment when each laser pulse (108  μJ) irradiated.

Figures 3(a) and 3(b) show an M-scan OCT image and the corresponding svOCT image of the bovine retina when 108-μJ energy per pulse irradiated. The M-scan OCT image shows the visible temporal signal variations induced by the laser pulse at the moment marked with white triangles. The signal variation increases the svOCT value. Figure 3(c) shows svOCT values averaged in ROI, and it shows a distinctive peak for each pulse irradiation.

**Fig. 4:**
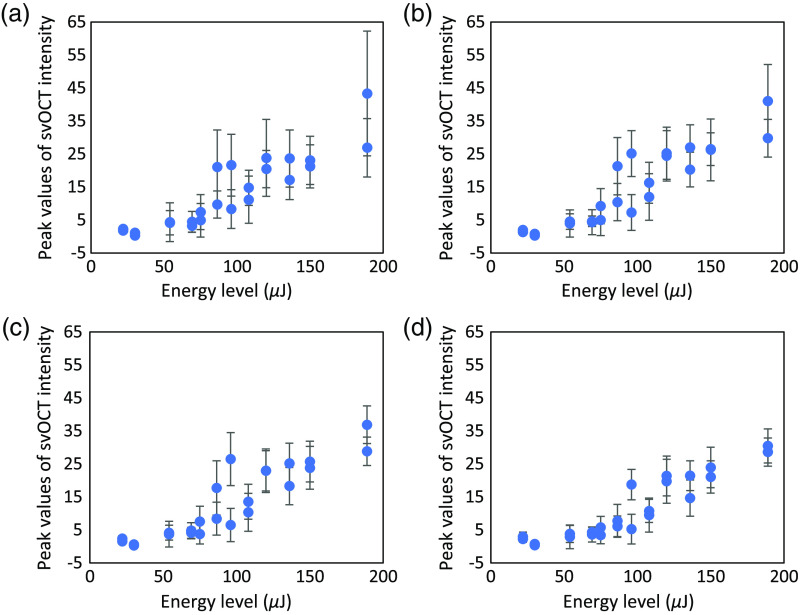
Mean (shapes) and standard deviation (error bar) of peak values of svOCT values averaged in ROI depending on laser pulse energy when window size N is (a) 2, (b) 5, (c) 10, and (d) 20.

To find appropriate N for calculating the svOCT image, we first tested different values of N and compared the average peak values of the svOCT spikes, the standard deviation of those peaks, and background noise level. Since the speckle variation induced by each laser pulse lasts for only around 50  μs, only five frames of the speckle variances show high signal while the rest show very low signal. Intuitively, if we choose a larger window size N, the lower portion of sv signal in the window will be significant, and it will decrease the overall value of the sv signal inside the window, i.e., the peak value of svOCT spikes. Furthermore, the variance of those peak values will also decrease. Figures 4(a)–4(d) show how the average peak values (shapes) and standard deviation (error bar) change depending on laser energy level when N is 2, 5, 10, and 20, respectively. We can see that, as expected, both the average peak value and the standard deviation decrease when N increases. Relative standard deviation, defined as the ratio of the standard deviation to the mean, was 0.53, 0.46, 0.43, and 0.46 for N of 2, 5, 10, and 20, respectively. Therefore, a mid-range of N values between 5 and 10 are suitable for calculating svOCT in terms of the precision and repeatability. In addition, note that the background noise level increases with increasing N. For N of 2, 5, 10, and 20, the average upper bound levels of background noise were 0.54±0.24, 0.74±0.31, 1.03±0.41, and 1.64±0.66, respectively. The background noise levels were bounded by μ+3σ upper limit, where μ was svOCT values averaged in ROI before or after laser irradiation and σ was the standard deviation. The increase in background noise was caused by the bulk motion of the sample and other environmental changes. Shorter integration time will decrease these effects and so will the smaller value of N. Considering both the effects of relative standard deviation and background noise levels, we choose N as 5.

**Fig. 5:**
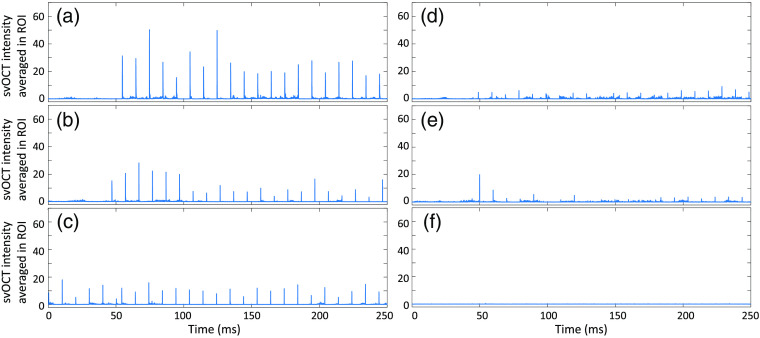
SvOCT values averaged in ROI when pulse laser energy is (a) 150, (b) 108, (c) 86, (d) 69, (e) 54, and (f) 30  μJ.

**Fig. 6:**
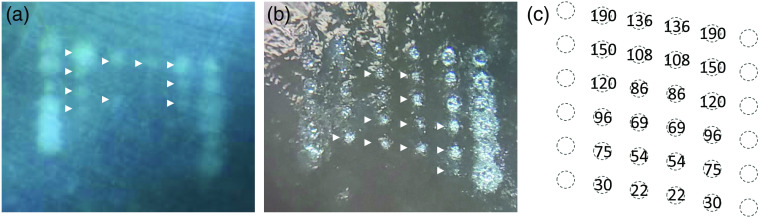
Microscopic image of the retina (a) before and (b) after peeling neural retinal layers off. (c) The energy level of treated spots.

Figure 5 shows svOCT values averaged in ROI depending on pulse laser energy when N is 5. Peak values increased with increasing pulse laser energy, and the distinctive peaks were observed when the laser pulse energy was as low as 54  μJ. Figures 6(a) and 6(b) show microscopic images of the treated spots before and after peeling upper neural retinal layers off, respectively. The energy level of each spot is shown in Fig. 6(c). The denaturation of neural retina can be observed as whitish spots (pointed by white triangles) in Fig. 6(a), but the lesions confined only to the RPE layer are invisible. The leftmost and rightmost columns are high-energy lesions marking pattern of the spots. In Fig. 6(b), lesions in the RPE layer (pointed by arrows) can be detected by peeling off the upper neural retinal layers.

**Fig. 7:**
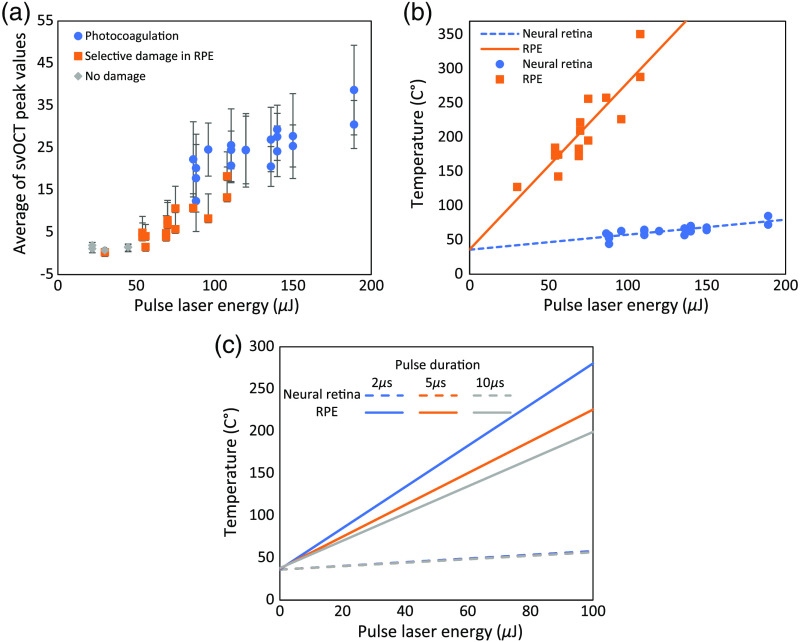
(a) Averaged peak values depending on pulse laser energy and damage range. (b) Simulated (lines) and estimated temperature from the svOCT intensity (shapes) at neural retina and RPE. (c) Simulated temperature at the neural retina and the RPE as a function of laser energy level for three pulse durations—2, 5, and 10  μs.

Average peak values of svOCT depend on the laser pulse energy; the result is summarized in Fig. 7(a). The data with blue circles indicate when the laser pulses induce a lesion in the upper neural layers in addition to inducing a lesion in the RPE layer [corresponding to the indicated spots in Fig. 6(a)]. The data represented by orange squares represent the cases when the lesions were induced only on the RPE layer, indicated by white triangle arrows in Fig. 6(b). The laser-induced lesion confined only to the RPE layer can be considered as a successful treatment. Fifteen peaks for each spot are averaged, and the standard deviation is shown by the error bar. As expected, it was difficult to define the threshold energy level that induces lesion only in the RPE layer. If the null hypothesis is defined as a successful treatment and the treatment is decided to be successful when the energy level is in the range from 41.9 to 92.1  μJ, which is determined by logistic regression, the type I error and the type II error were 26.7% and 25%, respectively. Compared with the energy level, the average peak values of svOCT showed a better correlation with the lesion creation. Average sv peak values ranged from 12.4 to 38.7 when the induced lesions were observed in both neural and RPE layers; sv values ranged from 0.3 to 18.3 when the lesion was confined only to the RPE layer. In the range of sv peak values from 0.7 to 1.9, no induced lesion was observed at all. When the treatment is decided to be successful with the average svOCT peak value between 1.88 and 15.3 based on logistic regression, the type I error and the type II error were 20% and 0.083%, which was better than the case when the threshold was set by the energy level. For dosimetry control, the method is designed to be used with a power ramped pulsed mode, in which laser energy increases linearly from pulse to pulse within one pulse train and automatically stops the next pulse irradiation when the svOCT peak values reached the predetermined threshold value.

In addition, the average peak value of svOCT intensities was correlated to the simulated temperature of the neural retina and melanosome surface in RPE. The linear regression of average peak values of svOCT on laser energy was calculated for photocoagulated lesions and selectively damaged lesions. Then, each of them was correlated to the simulated temperature of neural retina and RPE because the tissue damage process was different from each other. Since the simulated temperature of melanosome surface in the RPE was also linear to pulse laser energy, it was correlated to the average peak values of selectively damaged lesions as

$$T_{M}=124.5+12.4P,$$

where $T_{M}$ is the temperature of melanosome surface in RPE and P is the average peak values of svOCT. Figure 7(b) shows the simulated temperature (solid line) and estimated temperature (square shapes) from the average peak values of svOCT. Most of the estimated temperatures at RPE were higher than 150°C; the lowest temperature was 127.7°C. This is reasonable since the microvaporization is known to occur at around 150°C. Similarly, the average peak values of the photocoagulated lesions were correlated to the temperature of the neural retina and empirically fitted to a line as

$$T_{N}=25.5+1.5P.$$

Figure 7(b) shows that the temperature of the neural retina was mostly estimated to increase higher than 50°C when the neural retina was photocoagulated.

We also simulated temperatures of the neural retina and the melanosome surface in RPE as a function of laser energy level for three pulse durations—2, 5, and 10  μs, as shown in Fig. 7(c). The temperature of the neural retina does not change significantly, but the temperature of the melanosome surface in RPE decreases as pulse duration increases. The decrease in temperature can be explained by less heat confinement with longer pulse duration due to heat diffusion during irradiation.

## 4 Conclusion

It was shown that the SRT could be successfully monitored by the svOCT imaging system when integrated with the SRT system. We tested our system performance using *ex vivo* bovine eyes; the svOCT showed distinctive signal variation corresponding to each laser pulse irradiation. The signal variations were proportional to pulse energy levels, and it had a reliable correlation with the creation of lesion within the retina. The temperature at the neural retina and RPE was estimated by svOCT peak values using temperature simulation results, which was consistent with the observed lesion creation. However, we could have missed some minor tissue damages when assessing the photocoagulation and RPE cell damage from the microscopic images. Therefore, more studies that incorporate further analysis supported by histology or fluorescence microscopy would be needed to obtain a more accurate correlation between the svOCT signal and retinal damage range. In addition, we plan to perform *in vivo* studies using a live animal model to fully validate the utility of this method as an automatic dosimetry control in clinical SRT systems.
